# Recent trends in cancer mortality in the UK

**DOI:** 10.1038/sj.bjc.6602450

**Published:** 2005-03-22

**Authors:** R Doll, J Boreham

**Affiliations:** 1 Epidemiological Studies Unit, University of Oxford, Radcliffe Infirmary, Unit Harkness Building, OX2 6HE Oxford, UK

**Keywords:** cancer mortality, survival, UK Europe

## Abstract

Comparisons of survival rates of given diseases with different treatments or in different places often gave misleading results until the introduction of controlled trials. Recent reports of relatively low survival rates following the treatment of cancer in the UK compared to the rates in other countries, not based on controlled trials, may consequently be misleading. Their validity has, therefore, been tested by comparing the levels and trends in mortality – the ultimate criterion by which the success or failure of any system of care can be judged. For this purpose, rates and trends in rates over 20–50 years have been compared in five European countries of similar economic status (France, Italy, the Netherlands, Sweden, and the UK). The UK rates are not generally worse than those in the other countries and are sometimes better. Exceptions were cancer of the lung, large bowel, and breast, the first of which is explained by differences in the prevalence of smoking.

Comparisons of the incidence and mortality rates of a variety of diseases in different populations have provided important clues to their causation. The differences have often been large – sometimes several hundred fold for the incidence of different types of cancer – and there has been little difficulty in establishing that they were genuine. Comparison of national differences in survival are similarly useful to assess the adequacy and competence of medical care under different systems of organisation; but the differences between the results in different developed countries are unlikely to have been large and they are much more difficult to establish. It is nevertheless worrying when reports suggested that survival rates have been lower in the UK than in some other European countries (Sant *et al*, 2003). However, were the survival rates truly comparable?

We have long known how unreliable reports of differences in survival have been when different treatments have been used by different people or in different institutions and that reliable comparisons came to be made only when the principle of controlled trials was accepted. Unfortunately, that method is not available for testing the efficacy of treatment in whole countries, and we have to make do as best we can with what limited evidence is available.

Fortunately, the treatment of many types of cancer has become more effective in the last 20 years and we can at least see whether or not the trends in mortality have been equally favourable in the countries whose medical care we want to compare. For mortality rates in developed countries are reasonably reliable, although they can be occasionally distorted by artefacts and we need always to bear that possibility in mind when odd divergences unexpectedly occur. They are, of course, also affected by differences in incidence. These, however, are, for the most part, not large within Europe, ranging up to five-fold, and are unlikely to have had much effect on differences in the trends in mortality, apart from those for cancers closely related to smoking and the consumption of alcohol, most notably for cancer of the lung.

## METHODS

In this paper, I compare only the trends in mortality in a few selected countries. To compare with the UK, I have chosen four other countries in the European Union, without any prior knowledge of what the results would be, requiring only that the countries would be of roughly similar economic status: namely, France, Italy, The Netherlands, and Sweden. I would have chosen Germany, but the changes that occurred with the reunification of the East and the West made this impossible, as I wished to trace the trends over the whole of the last 50 years. Trends must be studied in both sexes, but it is not obvious what age groups are the most appropriate to compare. For studying the causes of disease these should certainly be relatively young, partly because incidence rates are less reliable in old age but primarily because rates in the young are more closely related to current conditions than the rates in the old. I doubt, however, if such rates would be appropriate for assessing the effects of treatment, as most cancers occur in the relatively old and one of the most important tests of the efficiency of a system of medical care is the provision it makes for the old. I have, therefore, compared the trends in mortality for all ages combined, standardising for age with the conventional European age distribution used by the World Health Organisation.

## COMPARISION OF TRENDS IN MORTALITY

### Two uncommon types of cancer

I examine first two relatively uncommon types of cancer for which new treatments have had a major effect on survival: namely cancer of the testis and Hodgkin's disease. For these two types I have examined only the rates since the mid-1970s when the new treatments came into common use.

**Figure 1:**
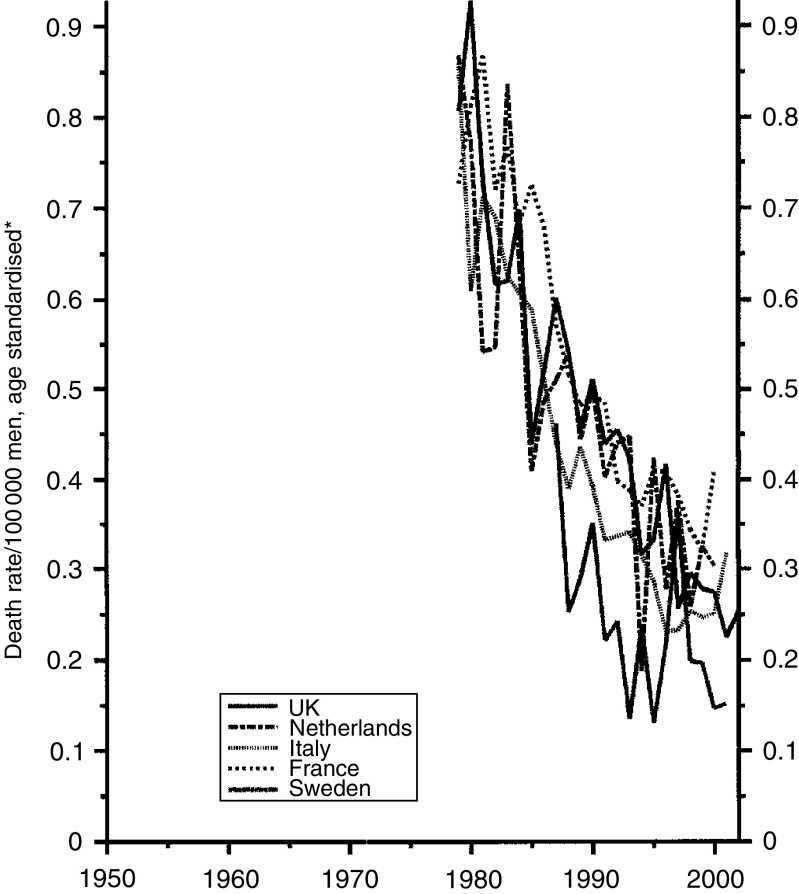
Five EU countries, 1979–2001, Males: testis cancer mortality at all ages. ^*^Annual rates per 100 000 men, standardised to conventional European age distribution. *Source*: WHO mortality and UN population estimates.

**Figure 2:**
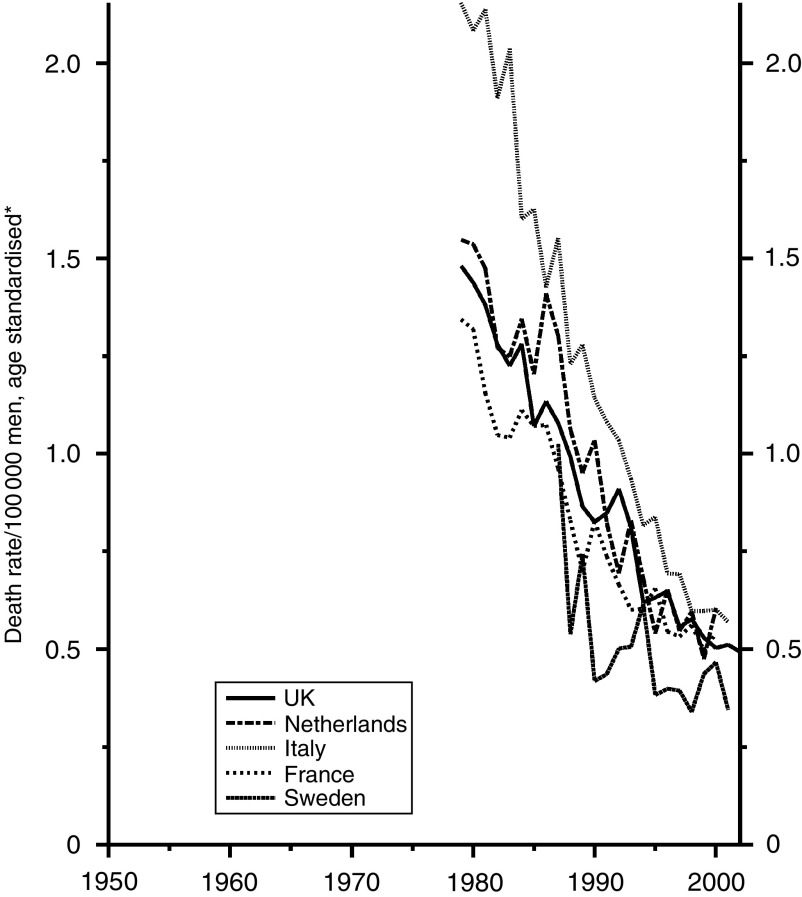
Five EU countries, 1979–2002, Males: Hodgkin's disease mortality at all ages. ^*^Annual rates per 100 000 men, standardised to conventional European age distribution. *Source*: WHO mortality and UN population estimates.

**Figure 3:**
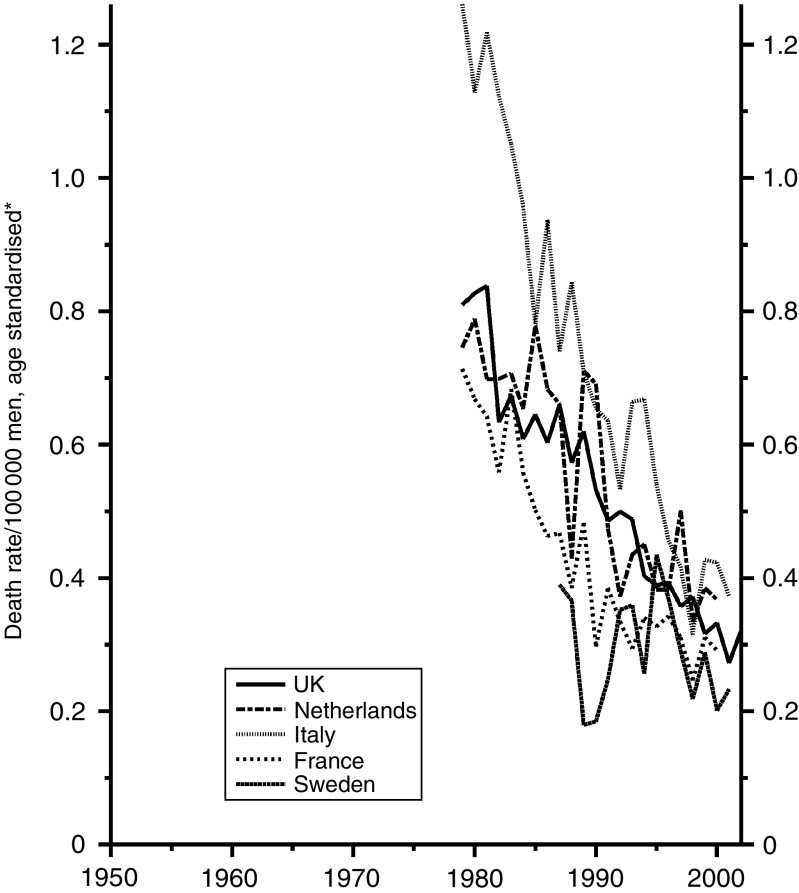
Five EU countries, 1979–2002, Females: Hodgkin's disease mortality at all ages. ^*^Annual rates per 100 000 women, standardised to conventional European age distribution. *Source*: WHO mortality and UN population estimates.

The data for testis cancer are shown in Figure 1. All five countries seem to have used the new treatments to much the same extent, with the UK trends in mortality near the middle. Figures 2 and 3 show the data for Hodgkin's disease separately for males and females. The reduction in mortality has again been much the same in all five countries since about 1985 with the British data near the middle. Before then the Italian data were exceptionally high due, according to La Vecchia (personal communication), to delay in adopting efficacious modern therapies in some parts of the country and partly to some national quirk of classification.

### Five common cancers

If now we consider the five most common cancers, there are two whose trends are dominated by changes in incidence: cancers of the stomach and lung. In both cases we can trace the trends since 1950.

#### Cancer of stomach

**Figure 4:**
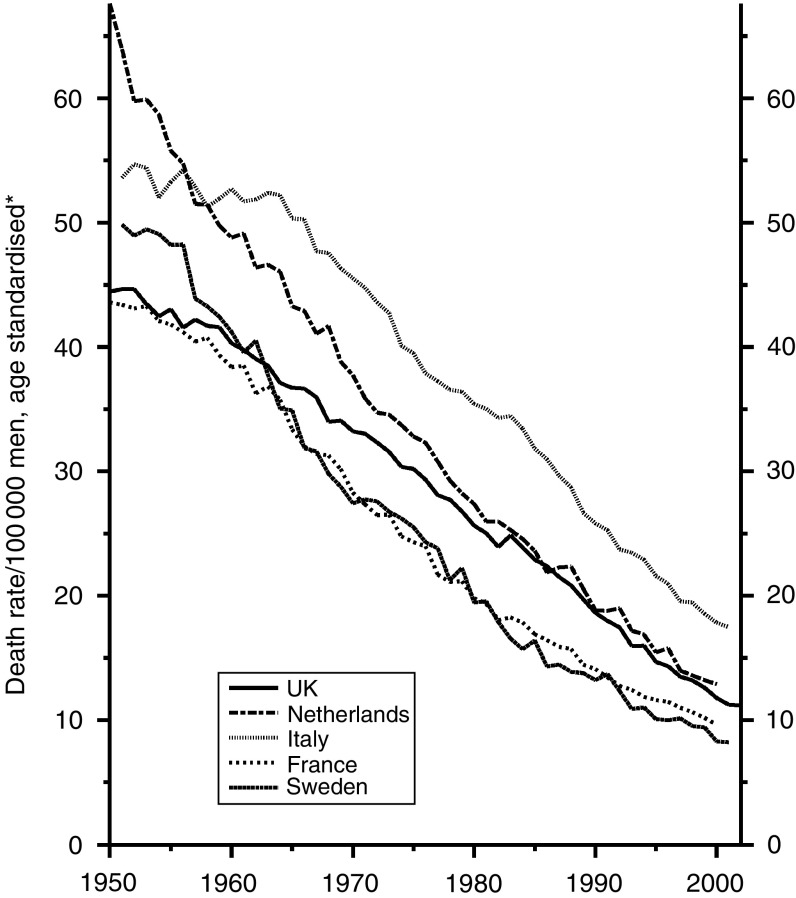
Five EU countries, 1950–2002, Males: stomach cancer mortality at all ages. ^*^Annual rates per 100 000 men, standardised to conventional European age distribution. *Source*: WHO mortality and UN population estimates.

With cancer of the stomach, the trends are the same in both sexes. Figure 4 shows the trends in men, with an astonishing reduction of about 80% over the period and with the UK data firmly in the middle. Whether improvements in treatment have played any material part may be open to doubt, but if they have there is no reason to suppose that they have been less in the UK than elsewhere – except perhaps than in Japan, whose data are not shown, where the incidence has been much higher than in Europe and intensive efforts have been made to treat the disease early in its history. Why there has been such a large and continued reduction in incidence is still uncertain. It seems to have been associated with the progressive increase in refrigeration and the abandonment of other forms of food preservation, the somewhat higher rate in Italy probably reflecting the continued use of salt preserved food in the mountainous areas. A reduction in the prevalence of gastric infection with *Helicobacter pylori* will also have contributed.

**Figure 5:**
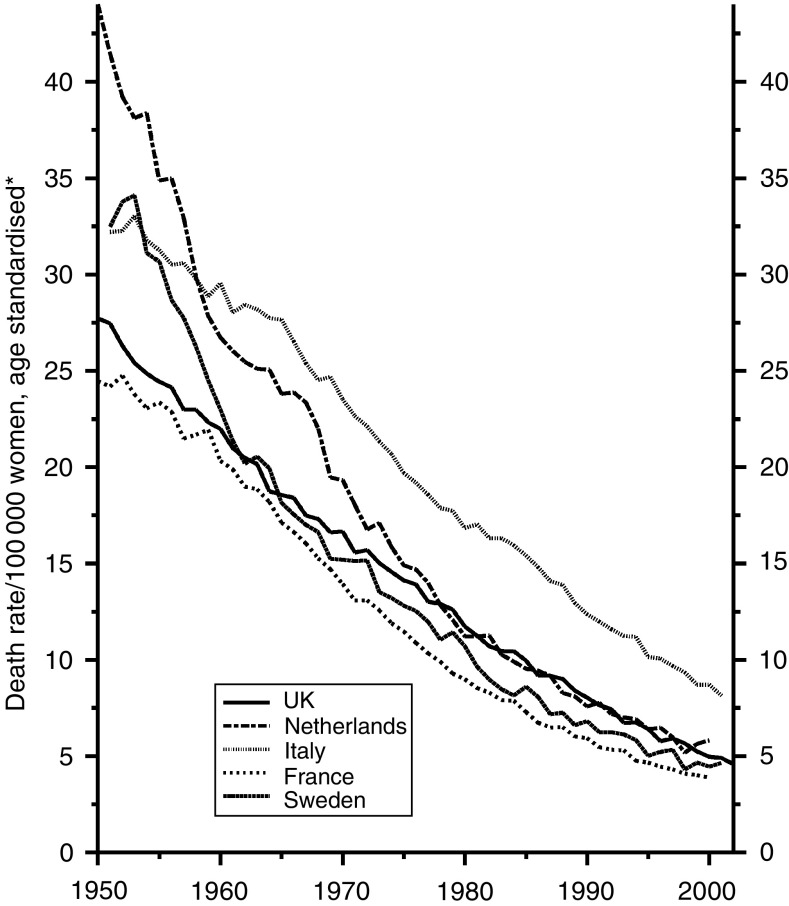
Five EU countries, 1950–2002, Females: stomach cancer mortality at all ages. ^*^Annual rates per 100 000 women, standardised to conventional European age distribution. *Source*: WHO mortality and UN population estimates.

Figure 5 shows that the trends have been almost identical in women.

#### Cancer of the lung

Unlike that for cancer of the stomach, the principal cause of cancer of the lung has varied in prevalence at different times in each country and in both sexes. There may have been some improvement in the outlook for some types of the disease in the hands of the ultraspecialist, but any such improvement can be detected only by controlled trials and is certainly too small to have made any detectable impact on the national mortality rate.

**Figure 6:**
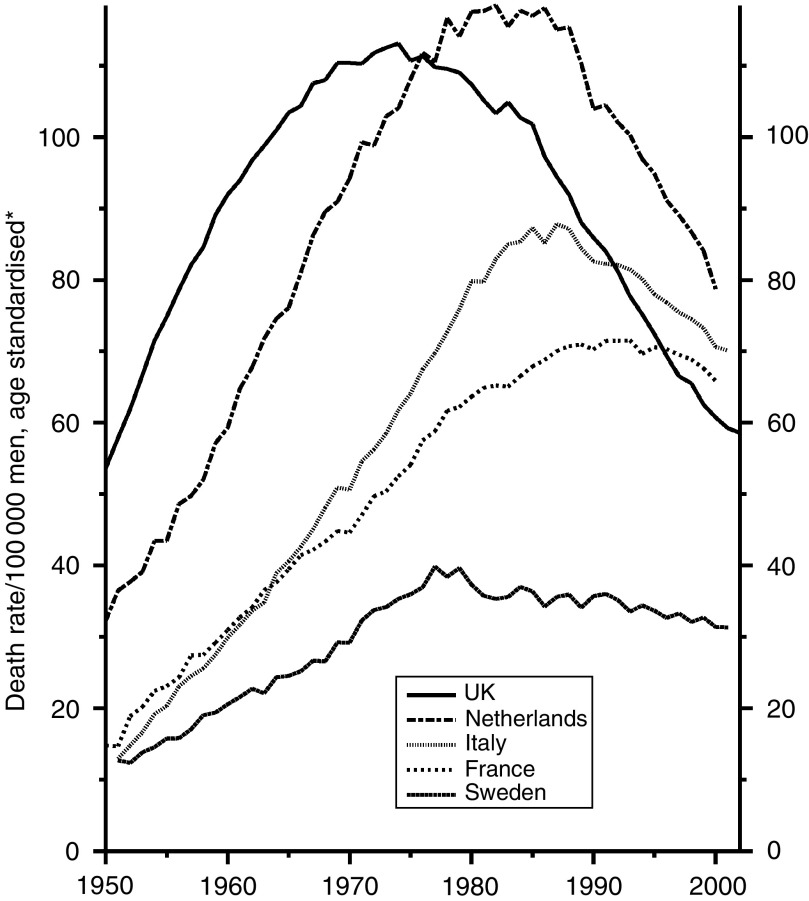
Five EU countries, 1950–2002, Males: lung cancer mortality at all ages. ^*^Annual rates per 100 000 men, standardised to conventional European age distribution. *Source*: WHO mortality and UN population estimates.

Figure 6 shows the trends over the last 50 years in men. Cigarette smoking became common first in the UK and the lung cancer mortality mounted steadily, at first in young men and then in the elderly until the time came that they had been smoking cigarettes from early in life. Separate examination by age and socioeconomic status shows that some reduction began in the young soon after the war, as a large price increase caused the young to reduce smoking and the very high tar content of the prewar cigarettes was, for some non-medical reason, reduced, while the most educated, who were most responsive to the spread of education, began to give up. It was not, however, until the early 1970s when the media were themselves convinced of the importance of the relationship and gave a clear message in the press and on the television that increasingly large numbers of smokers stopped smoking and a decline began to be seen progressively at all ages. The adoption and decline of cigarette smoking followed later in most other countries, but the habit never became really popular among Swedish men.

**Figure 7:**
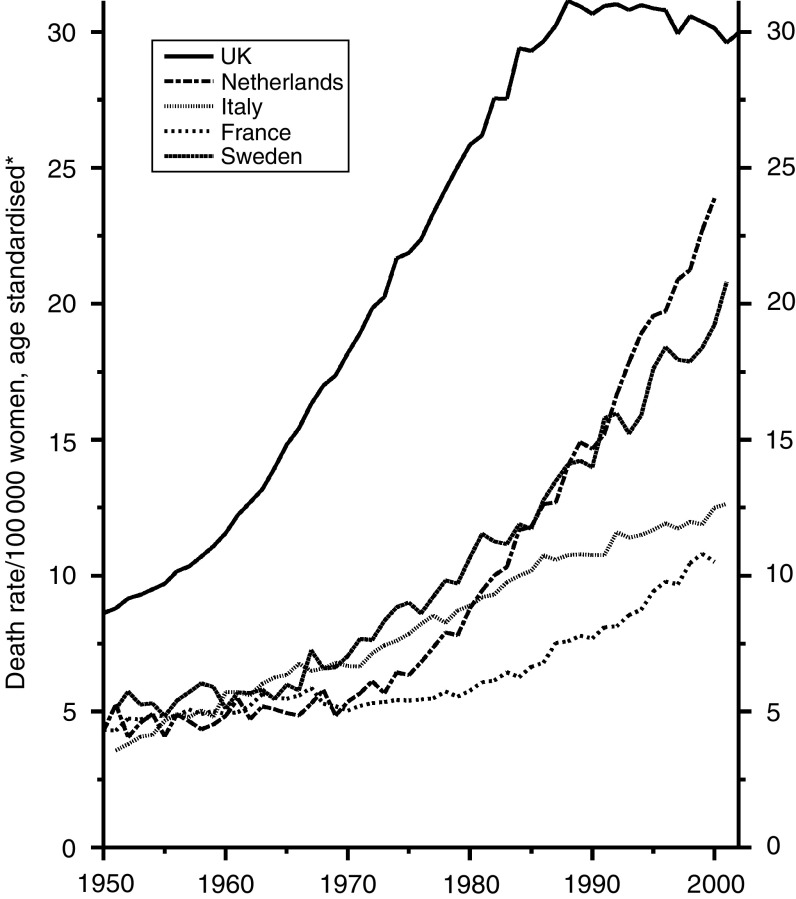
Five EU countries, 1950–2002, Females: lung cancer mortality at all ages. ^*^Annual rates per 100 000 women, standardised to conventional European age distribution. *Source*: WHO mortality and UN population estimates.

Figure 7, which is on a different scale, reflects the later development of smoking in women, in whom the risk in the four countries other than the UK is still rising.

**Figure 8:**
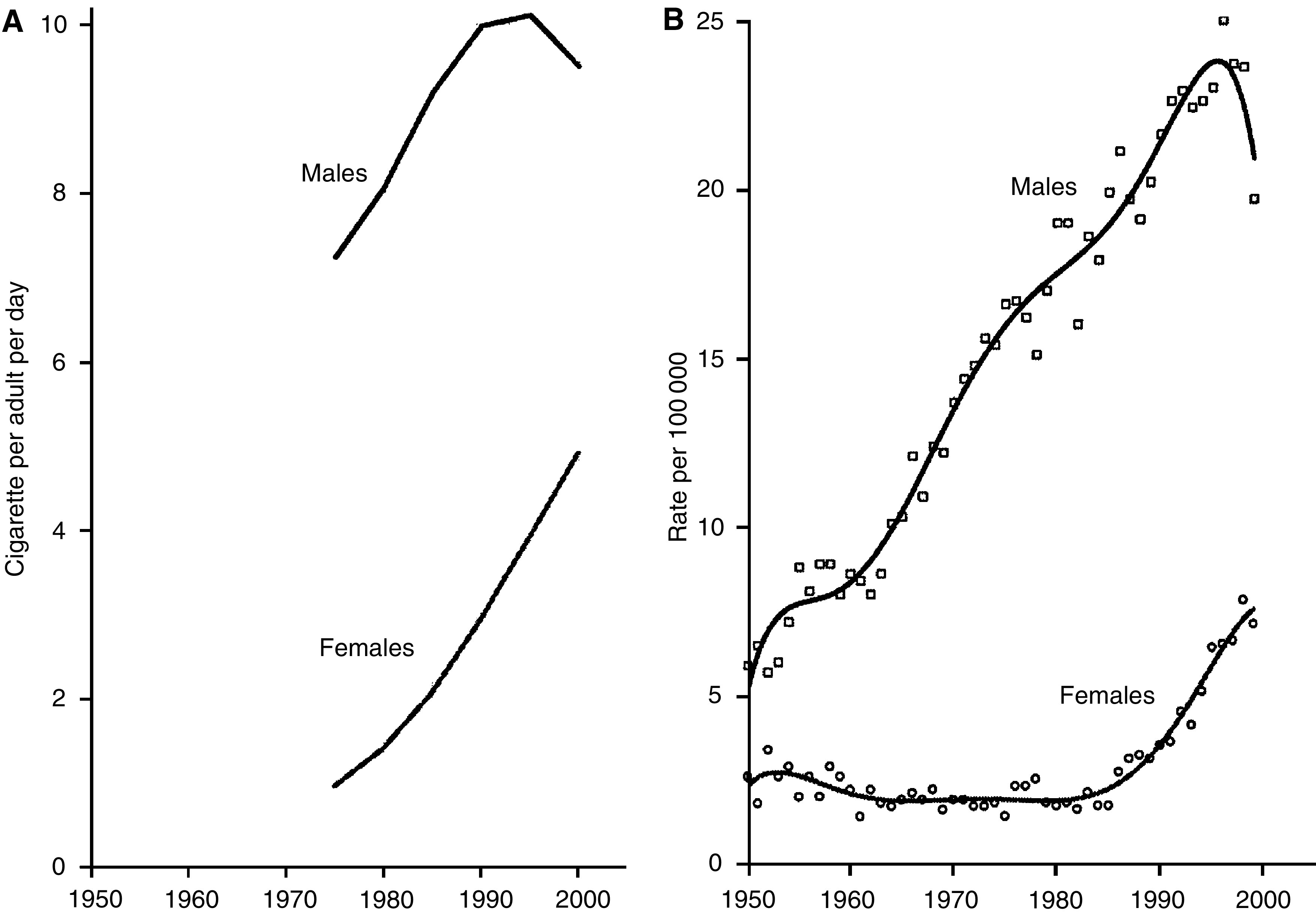
Trends in lung cancer at ages 40–44 and past cigarette smoking in men and women in France 1950–2000. (**A**) Average number of cigarettes per day between age 15 and 42.5, population aged 40–44. (**B**) Lung cancer death rate, population aged 40–44.

The close association between risk of lung cancer and the amount smoked is beautifully illustrated by the demonstration of the relationship between the risk in young men in France and their estimated lifetime consumption of cigarettes shown in Figure 8 (Hill and Laplanche, personal communication).

#### Cancer of the breast

**Figure 9:**
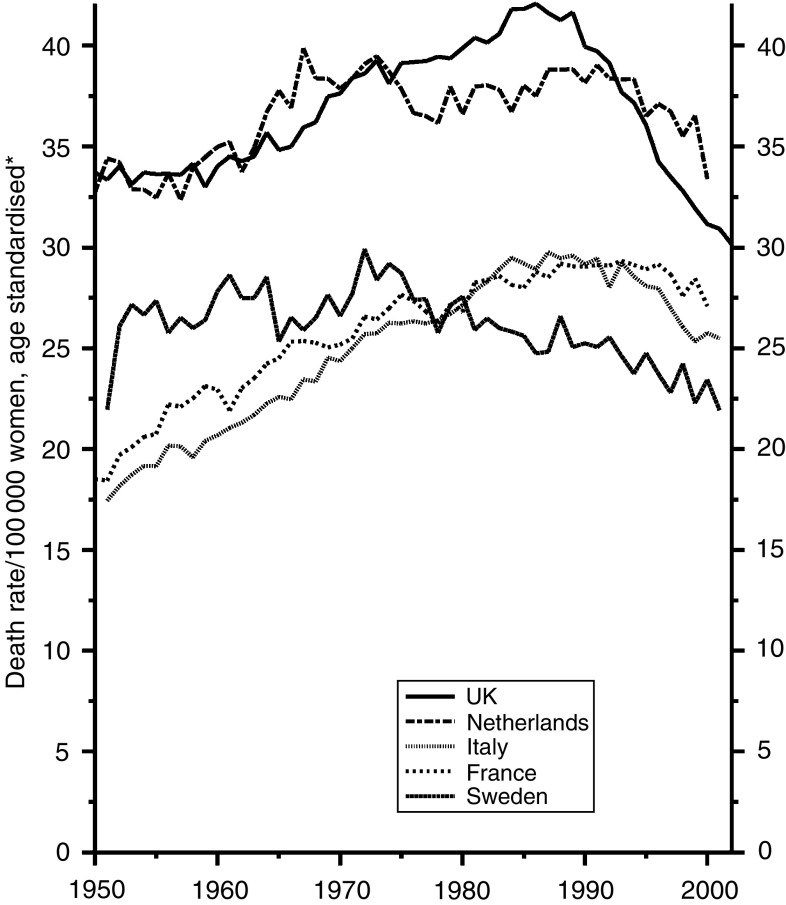
Five EU countries, 1950–2002, Females: breast cancer mortality at all ages. ^*^Annual rates per 100 000 women, standardised to conventional European age distribution. *Source*: WHO mortality and UN population estimates.

Two other types of cancer are more pertinent for the purpose of this review as the recent trends in their mortality have been affected principally by the efficiency of treatment: namely, cancers of the breast and prostate. The trends for the former are shown in Figure 9. The hump in UK mortality between 1984 and 1992 is an artefact due to a change in the rule allocating the underlying cause of death when cancer was mentioned on the death certificate as an associated cause. The Registrar-General for England and Wales, who had changed the rule, hoped that other countries would follow suit; but they did not and he quickly returned to the old rules laid down by the World Health Organisation. Whether the relatively high mortality in the Netherlands and the UK between 1950 and 1990 reflected a higher incidence or less effective treatment is a moot point. There is some evidence that the incidence has been unusually high in the Netherlands, but not in the UK (International Agency for Research on Cancer, 1992). Whatever the explanation, however, the sharp drop in mortality since 1990 has brought the UK rate close to those of Italy and France. The fall is principally due to the increased use of chemotherapy and particularly the intensive and prolonged use of tamoxifen, which was uncommon in the UK until the combined results of the Early Breast Cancer Trialists Collaborative Group (1988) were publicised. Some contribution is also likely to have come from increased specialist care and the more extensive use of mammography, but the latter would not have affected all the age groups in which a reduction in mortality has been seen.

#### Cancer of the prostate

**Figure 10:**
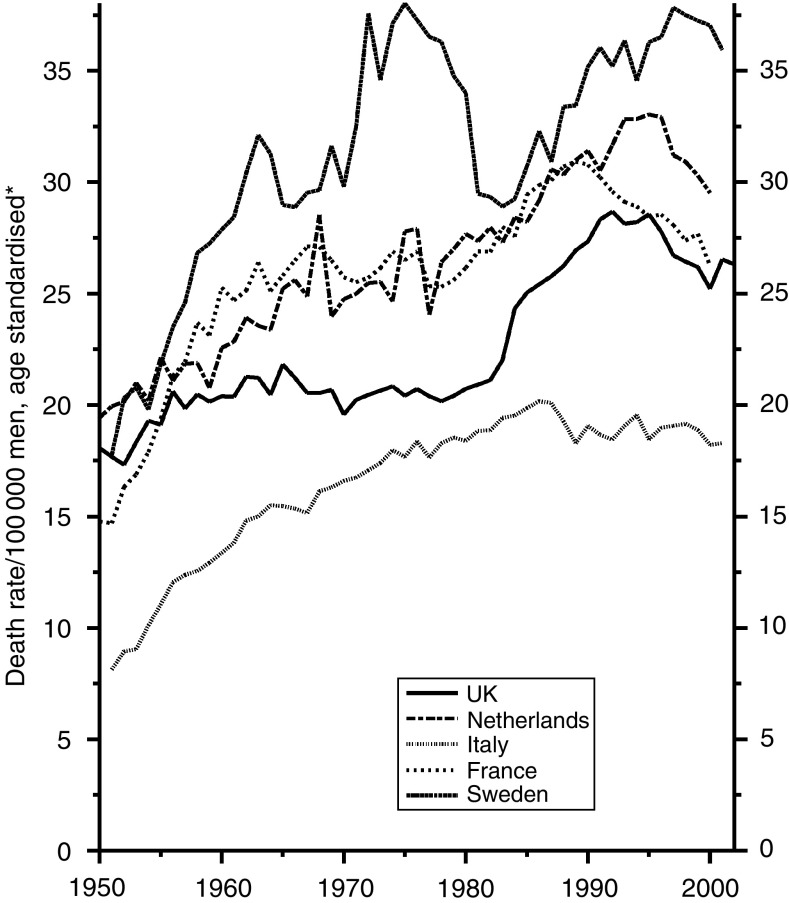
Five EU countries, 1950–2002, Males: prostate cancer mortality at all ages. ^*^Annual rates per 100 000 men, standardised to conventional European age distribution. *Source*: WHO mortality and UN population estimates.

Figure 10 shows the trends for prostate cancer. They are truly remarkable. We see again the small artificial hump in the British data between 1984 and 1992, but are also faced with the extraordinary hump in the Swedish data in the 1970s. According to Professor Ekbom (personal communication), the changes that took place in and after the 1950s can be largely explained by effects on death certification of major changes in the method of medical re-imbursement, the frequency of biopsy, and methods of operation, affected possibly in part by PSA measurements.

Mortality rates in France, the Netherlands, and the UK all showed a substantial rise in the 1980s, before there began to be a fall due almost certainly to chemotherapy, which is shown most clearly, as with breast cancer, by the data for relatively young age groups in which treatment has been more aggressive. However, whatever the explanation for these complex trends there is nothing to suggest that British survival rates have been unusually poor.

#### Colorectal cancer

**Figure 11:**
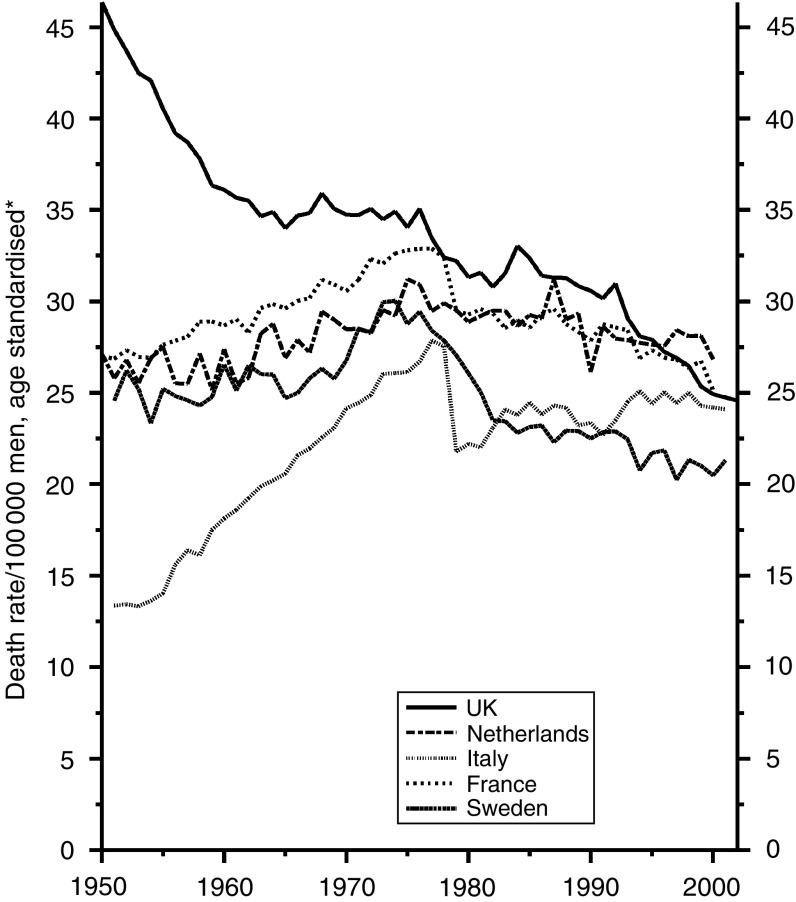
Five EU countries, 1950–2002, Males: colorectal cancer mortality at all ages. ^*^Annual rates per 100 000 men, standardised to conventional European age distribution. *Source*: WHO mortality and UN population estimates.

**Figure 12:**
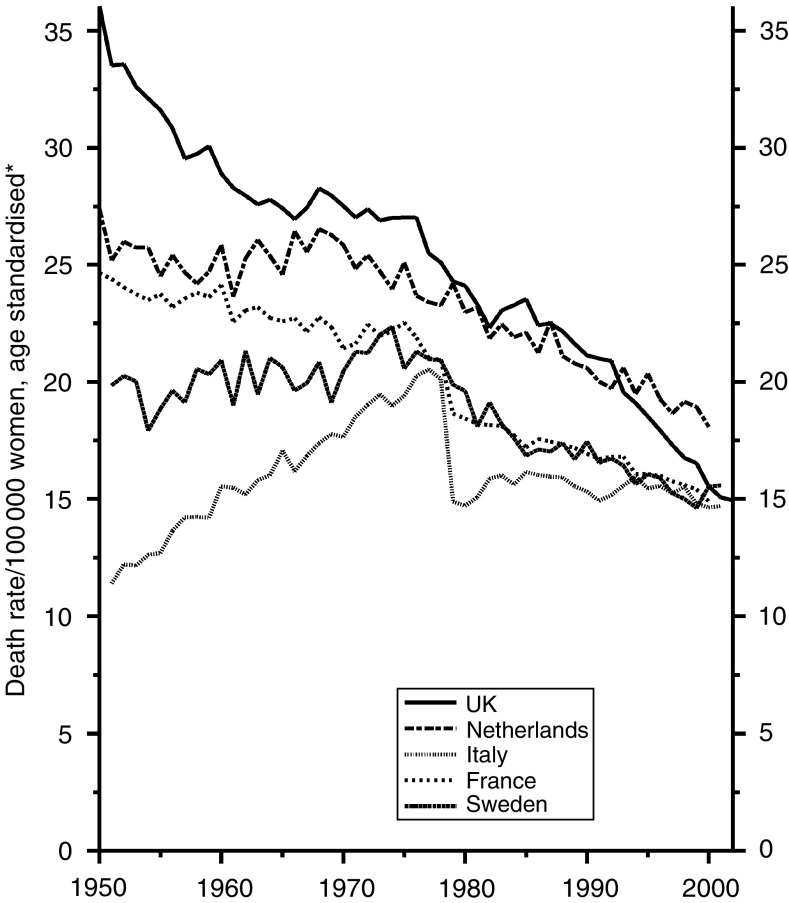
Five EU countries, 1950–2002, Females: colorectal cancer mortality at all ages. ^*^Annual rates per 100 000 women, standardised to conventional European age distribution. *Source*: WHO mortality and UN population estimates.

The last of the major causes for which I have sought specific figures is cancer of the large bowel. The trends are shown in Figures 11 and 12. I have no explanation for the low rates in Italy before 1970, but the sharp drop in mortality in the late 1970s can be explained as an artefact resulting from the belated recognition that cancers of the small bowel and of the intestine unspecified should not have been included with the specific data for cancers of the large bowel. For the purpose of the present review it is notable that the rates in all five countries are now much the same with the UK rates firmly in the middle.

### All cancers other than lung cancer

**Figure 13:**
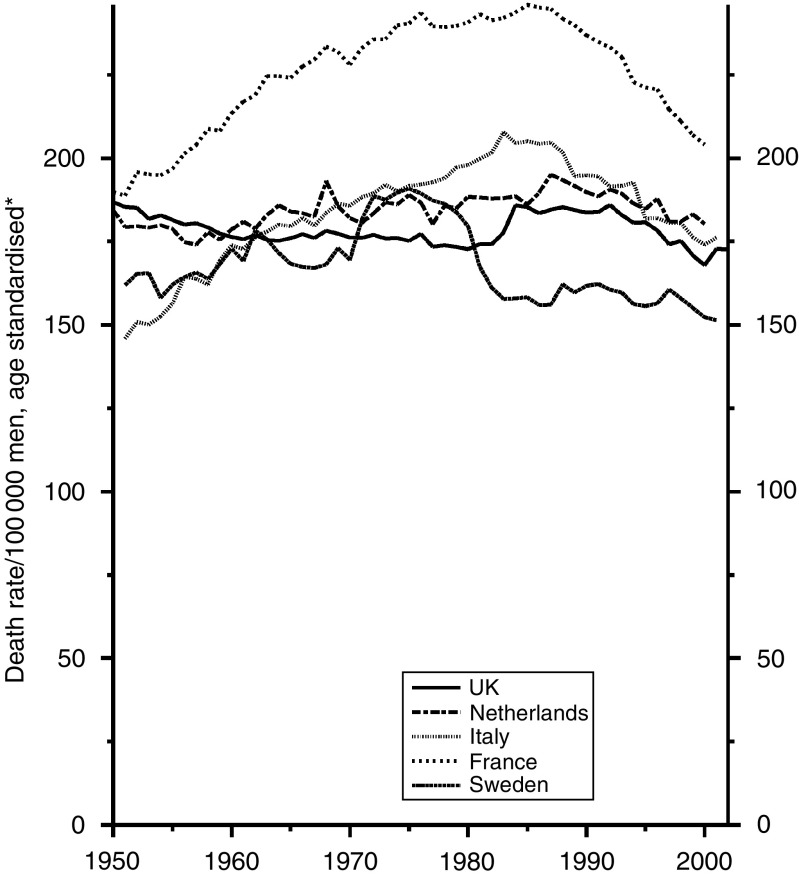
Five EU countries, 1950–2002, Males: all cancer (excl. lung) mortality at all ages. ^*^Annual rates per 100 000 men, standardised to conventional European age distribution. *Source*: WHO mortality and UN population estimates.

**Figure 14:**
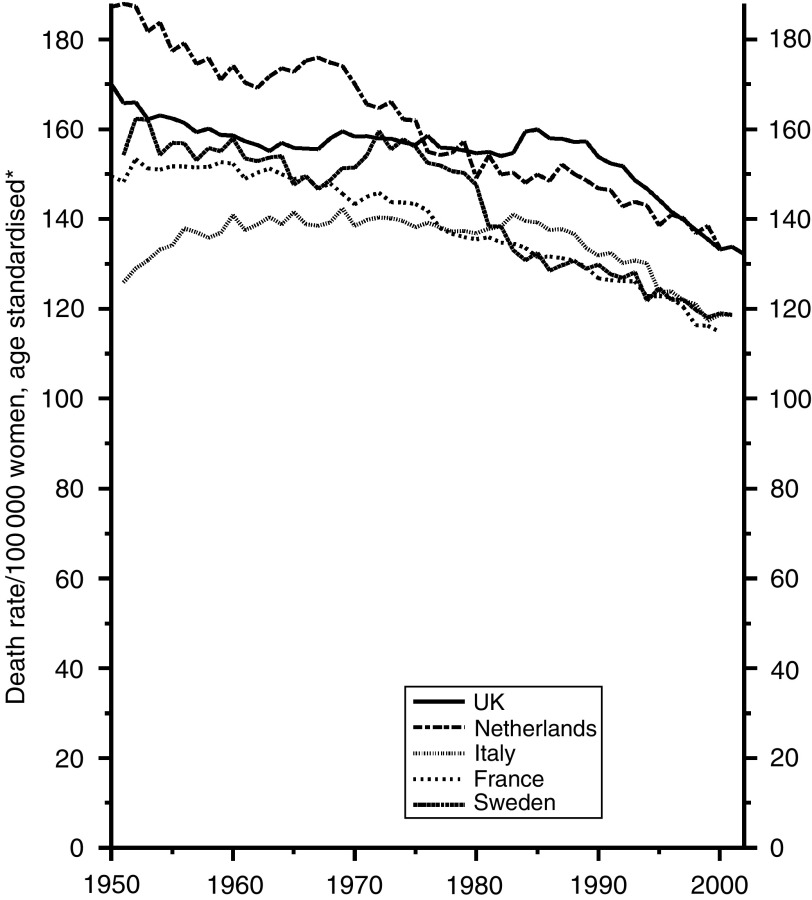
Five EU countries, 1950–2002, Females: all cancer (excl. lung) mortality at all ages. ^*^Annual rates per 100 000 women, standardised to conventional European age distribution. *Source*: WHO mortality and UN population estimates.

For all cancers combined the trends are materially affected by those for lung cancer, which have little bearing on the efficiency of diagnosis and treatment. I have examined, therefore, only the trends for all cancers other than lung cancer, which are shown for men in Figure 13 and for women in Figure 14.

Those for men show the artificial hump in Sweden in the 1970s and consistently high figures for France, reducing substantially since 1985, which may be due to the recent reduction in the previously high consumption of alcohol and its consequent effect on the mortality from cancers of the mouth, pharynx, larynx, oesophagus and liver. The UK figures have been the lowest but one since the 1960s and show no tendency to depart from that position. The trends for women, though consistently downward for all five countries since the mid-1980s, are less satisfactory for the UK. The current rates are slightly higher than those for Italy, France and Sweden and equal highest with those for the Netherlands, due possibly to the prevalence of smoking by women, as smoking contributes to many types of cancer other than cancer of the lung.

## CONCLUSION

The British rates for the mortality from cancer, which is the ultimate criterion by which the success or failure of any system of care and therapy for patients with cancer has to be judged, are not generally worse than those in other economically comparable European countries, and, indeed, are sometimes better. This was not so for the trends in breast and colorectal cancer before 1990 and the recent decline in the mortality from both these diseases may reflect improvements in treatment and consequently in survival.
